# Within-group relationships and lack of social enhancement during object manipulation in captive Goffin’s cockatoos (*Cacatua goffiniana*)

**DOI:** 10.3758/s13420-016-0235-0

**Published:** 2016-07-12

**Authors:** B. Szabo, T. Bugnyar, A. M. I. Auersperg

**Affiliations:** 1 Department of Biological Sciences, Macquarie University, 209 Culloden Road, Building W19A, Marsfield, NSW 2122 Australia; 2 Department of Cognitive Biology, University of Vienna, Althanstr. 14, Vienna, 1090 Austria; 3 Comparative Cognition Unit, Messerli Research Institute, University of Veterinary Medicine, Medical University of Vienna, University of Vienna, Veterinärplatz 1, Vienna, 1210 Austria

**Keywords:** Avian cognition, Social learning, Parrot, Dominance hierarchy

## Abstract

Different types of social relationships can influence individual learning strategies in structured groups of animals. Studies on a number of avian species have suggested that local and/or stimulus enhancement are important ingredients of the respective species’ exploration modes. Our aim was to identify the role of enhancement during object manipulation in different social contexts. We used focal observations to identify a linear dominance hierarchy as well as affiliative relationships between individuals in a group of 14 Goffin’s cockatoos (*Cacatua goffiniana*, formerly *goffini*). Thereafter, in an unrewarded object choice task, several pairs of subjects were tested for a possible influence of social enhancement (local vs. stimulus) in three conditions: dominance, affiliation, and kinship. Our results suggest strong individual biases. Whereas previous studies on ravens and kea had indicated that enhancement in a non-food-related task was influenced by the social relationship between a demonstrator and an observer (affiliated – nonaffiliated), we found no such effects in our study group. In this context, Goffin’s cockatoos’ object learning seems to take place more on an individual level, despite their generally high motivation to manipulate nonfood items.

**Electronic supplementary material:**

The online version of this article (doi:10.3758/s13420-016-0235-0) contains supplementary material, which is available to authorized users.

To regulate access to resources and mates (Ficken, Weise, & Popp, 1990; Hinde, 1976), many group-living animals develop dominance hierarchies (Ficken et al., 1990; Hinde, 1976; Humphrey, 1976). The hierarchical position is usually established by a repeated exchange of agonistic interactions (Drews, 1993; Kappeler, 2006; Paz-y-Mino et al. 2004), or is even inherited from the parents (Bergstrom & Fedigan, 2010) or transferred from mating partners (Lorenz, 1935; Röell, 1978).

In addition to agonistic interactions, social groups can also be characterized by affiliative relationships between both related and unrelated individuals (e.g., Hinde, 1976). Affiliates spend more time in close proximity and tend to show high levels of reciprocal socio-positive behaviors (Bonnie & de Waal, 2006; Schwab, Bugnyar, Schloegl, & Kotrschal, 2008). Individuals profit from such relationships by gaining support in agonistic interactions, sharing valuable information or resources (Fraser & Bugnyar, 2010), and in the case of kin, may increase their inclusive fitness (Hamilton, 1964a, 1964b). Keeping track of the identities and social relationships of all members within a social group and adjusting behavioral responses accordingly may represent a high cognitive load to some species (see the summaries in Byrne, 1989; Humphrey, 1976).

During interactions, information may be transferred from one individual to another, a process called *social learning* (Heyes, 1994). Social learning is considered advantageous particularly in uncertain situations, when an individual is confronted with unfamiliar food or predators (Laland, 2004; Zentall, 2012). In frequently changing environments, however, social learning can lead to misinformation, since any advantageous information may quickly be outdated (Giraldeau et al., 2002; Kendal, Coolen, & Laland, 2009a). Hence, to be adaptive, social learning should be used selectively (Galef, 1995); that is, when individually sampling the environment bears high temporal and energy costs and/or a high risk of injury (Bonnie & Earley, 2007; Giraldeau et al., 2002; Kendal et al., 2009a; Kendal, Giraldeau, & Laland, 2009b), individuals should revert to learning from conspecifics (Arbilly, Weissman, Feldman, & Grodzinski, 2014; Boyd & Richerson, 1988; Galef, 1995; Giraldeau, Valone, & Templeton, 2002; Kendal, Coolen, van Bergen, & Laland, 2005; Laland, 2004; Zentall, 2012).

It is well-known that humans behave according to the norms dictated by their social environment (Merton & Rossi, 1949, cited by Bearden & Etzel, 1982). The phenomenon that humans are influenced by the decisions and opinions of others (Bandura, 1986) is used by marketers for designing advertisements (Bearden & Etzel, 1982). Coussi-Korbel and Fragaszy (1995) proposed a similar influence in animals—namely, that the behavior patterns of one individual will have an influence on others, depending on their relationship (Katzir, 1982; Stöwe et al., 2006), and should alter their decisions with respect to the identity of a demonstrator during social learning. Expanding on this line of thought, Laland (2004) and Rendell et al. (2011) described a number of strategies concerning when social learning occurs and from whom individuals should learn.

On the basis of the type of information gained from the observation of a demonstrator (e.g., single stimulus, location, stimulus–stimulus relationship, affordances, or complex motor tasks), social learning is divided into subcategories. Among these, stimulus and local enhancement are assumed to be two of its simplest but most widespread forms (Coussi-Korbel & Fragaszy, 1995; Hoppitt & Laland, 2008). According to Hoppitt and Laland, *stimulus* enhancement occurs when the observation of a demonstrator (or its products) exposes the observer to a single stimulus at a time *t*_1_ and that single stimulus exposure effects a change in that observer’s behavior, at a second time *t*_2_. In contrast, *local* enhancement occurs when, after or during a demonstrator’s presence at, or interaction with objects at, a particular location, an observer is more likely to visit or interact with objects at that location (Hoppitt & Laland, 2008). In accordance with these definitions, an increase in stimulus handling time can be predicted if enhancement occurs.

By allowing frequent and stable physical proximity and/or by directing a conspecific’s attention to specific stimuli, social dynamics could favor different forms of social learning (Coussi-Korbel & Fragaszy, 1995). Only a small number of studies have focused on the influence of these dynamics on different types of enhancement, and even fewer have done so in a nonfeeding context. Although enhancement may occur in both feeding and nonfeeding situations, independent of the benefits, a lack of enhancement in one context might not necessarily indicate a lack thereof in the other, or a general lack of the ability to use any form of social learning. The results of previous studies in birds have demonstrated a surprising variety of social influence on enhancement in three corvids (*Corvus corax*: Schwab, Bugnyar & Kotrschal 2008a; Heyse, 2012; *Corvus corone corone & C. c. cornix*: Miller, Schiestl, Whiten, Schwab, & Bugnyar, 2014; *Corvus monedula*: Schwab, Bugnyar, & Kotrschal, 2008), as well as in New Zealand’s kea parrots (*Nestor notabilis*: Heyse, 2012; see Table 1).

**Table 1:** Comparative summary of the methodologies used within the presented sample of avian literature testing for social enhancement: Study, species, sample size, age of subjects., presentation of objects during the demonstration and the test phase, the subjects prior familiarity with the used objects, type of object presentation, food/no-food involved, the appearance of the introduced objects, the type of contact between the demonstrator and the subject during the demonstration and the test phase, if demonstrators participated as subjects during the study and vice versa and if the arrangement of the setup was observable to the subjects

				Presentation of Objects				Food Involve-ment		Contact			Experimenter Induced Enhancement
Study	Species	*N*	Subject Age	Demon-stration	Test	Object Familiarity	Object Use		Object Appearance	Demon-stration	Test	Role Switch	
Schwab, Bugnyar, & Kotrschal (2008)	*Corvus monedula*	20	juvenile	one object (=target)	5 objects including target	unfamiliar	once by pair	no	grouped by shape/size, target identical	visual contact	no contact	yes	possible
	*Corvus monedula*	20	juvenile/ adult	2 distinctly colored boxes	2 distinctly colored boxes	familiar	once by pair	yes	grouped by color, identical	visual contact	no contact	yes	possible
Schwab, Bugnyar, Schloegl, & Kotrschal (2008)	*Corvus corax*	12	juvenile	one object (=target)	5 objects including target	unfamiliar	once by observer	no	grouped by limited dissimilarity, target identical	visual contact	no contact	yes	possible
Miller et al. (2014)	*C. c. cornix*	115	juvenile/ adult	2 pairs	2 pairs	familiar	repeatedly	partly	pair of same size & shape, different color	physical contact	physical contact	possible	possible
	*C. c. corone*	115	juvenile/ adult	2 pairs	2 pairs	familiar	repeatedly	partly	pair of same size & shape, different color	physical contact	physical contact	possible	possible
Heyse (2012)	*C. corax*	11	juvenile/ adult	4 pairs	4 pairs	unfamiliar	repeatedly	no	objects in a pair of different complexity, 4 pairs identical	physical contact	physical contact	possible	possible
	*Nestor notabilis*	11	juvenile/ adult	4 pairs	4 pairs	unfamiliar	repeatedly	no	objects in a pair of different complexity, 4 pairs identical	physical contact	physical contact	possible	possible
Current study	*Cackatua goffiniana*	14	juvenile/ adult	2 pairs	2 pairs	partly familiar	once by observer	no	first & third similar, second & fourth similar, not identical	visual contact	no contact	yes	possible

Stimulus enhancement was demonstrated by Schwab, Bugnyar, Schloegl, and Kotrschal (2008) in sibling and nonsibling pairs of juvenile ravens. First, subjects watched a demonstrator manipulate a nonfood object in an adjacent room. Thereafter, the observer was confronted with a set of five objects, including the target object that the demonstrator had manipulated just moments before. Subjects within sibling pairs manipulated the target object significantly longer than the other four objects, whereas nonrelated birds showed no enhancement. Using the same paradigm, Schwab, Bugnyar, and Kotrschal (2008) showed that juvenile jackdaws were influenced by neither a sibling’s nor a nonsibling’s choice. However, two additional food-related experiments demonstrated enhancement in juvenile and adult jackdaws observing nonsiblings/nonpair mates feeding from one of two distinctively colored boxes containing mealworms. Controls for side preferences indicated that the mechanism was stimulus rather than local enhancement. These results suggest an impact of species’ feeding ecology on enhancement. Ravens use food caching and develop cache protection or pilfering strategies during social object play and play-caching (Bugnyar et al. 2007). Noncaching corvids such as jackdaws may, therefore, be less attentive to the (nonfood) object manipulations of others. On the basis of these findings, food seems to have greater influence on noncaching corvids.

Miller et al. (2014) studied social enhancement in free-ranging carrion crows by providing groups of crows with two pairs of objects. Observers were more likely to interact with an object at the same location as a demonstrator than with a second, identical object 2 m away. In half of the sessions, a piece of bread was placed underneath the objects. The authors state that co-feeding occurred more often than affiliative and agonistic behaviors and that the animals were very tolerant of conspecifics feeding next to them. It is therefore not surprising that local enhancement was detected, since tolerating conspecifics in close proximity is a necessary prerequisite (Coussi-Korbel & Fragaszy, 1995).

Another study using a group setup tested social enhancement in ravens and kea (Heyse, 2012). Subjects were presented with four pairs of items, among which they could choose freely. Generally, both species showed stimulus enhancement most frequently, and it was favored by affiliated subjects. Additionally, higher-ranking ravens showed more local enhancement. Rank-dependent resource access might have enabled them to use local enhancement as a learning mechanism (Laland, 2004), whereas affiliation could lead to an increased awareness of a conspecific’s actions, and attention might selectively be directed to specific stimuli, making stimulus enhancement a likely learning mechanism (Coussi-Korbel & Fragaszy, 1995).

Goffin’s cockatoos (*Cacatua goffiniana;* formerly *goffini*) are generalist parrots endemic to the Tanimbar Islands in Indonesia. They live in social groups (*N* < 100), mated pairs, or family groups in tropical dry forests (Cahyadin, Jepson, & Manoppo, 1994; Jepson, Brickle, & Chayadin, 2001). In captivity, these cockatoos show a wide range of social interactions, as well as complex and structured object play and manipulative exploration behavior (Auersperg, Oswald, et al. 2014; Auersperg et al., 2015). Behavioral observations have revealed that they spend most of the day manipulating a variety of different objects (Auersperg et al., 2015; Szabo, 2013) and, on the basis of these findings, we decided to apply an unrewarded object choice task to study enhancement. Furthermore, this species had previously demonstrated high levels of performance in a number of cognitive tasks, such as impulse control (Auersperg, Laumer, et al. 2012), sequential problem solving (Auersperg, Kacelnik, & von Bayern, 2013), Piagetian object permanence (Auersperg, Szabo, von Bayern, & Bugnyar, 2014), and the capacity to innovate tool use as a solution to a novel problem (Auersperg, Szabo, von Bayern, & Kacelnik, 2012). Notably, Goffin’s cockatoos are able to socially transmit information to conspecifics in a foraging task involving the use of tools (Auersperg, von Bayern, et al., 2014). Enhancement therefore seems to play a role in at least some foraging tasks (Auersperg, von Bayern, et al., 2014); it is, however, unclear which enhancement processes (local vs. stimulus) are common, and to what extent enhancement is influenced by their social relationship to the respective demonstrators. On the basis of our current state of knowledge, the Goffin is an opportunist/generalist species that explores a wide range of visually distinct resources (unpublished field data); therefore, stimulus enhancement seems most likely as an enhancement mechanism. Furthermore, like many other *Cacatua* species, they show high levels of aggression toward nonaffiliated individuals (Forshaw & Cooper, 2003). To avoid aggression, attention might be directed selectively toward affiliates and subordinates, rather than dominant conspecifics. However, the opposite might be the case, because dominant individuals generally have better access to desired or limited resources, and are therefore a more reliable source of information (Laland, 2004). Gaining a more detailed insight into how social relationships influence their object exploration would represent an important next step to improve our understanding of the role of social learning in the technical abilities of this avian model.

Our aim was first to determine the social structure of our available group, by evaluating the type of dominance hierarchy as well as possible affiliative relationships between individuals, and to create pairs in three relationship categories (determined by rank, unrelated affiliation and relatedness). Thereafter, we applied a simple social-learning experiment testing for enhancement in a non-food-related object choice task.

## Method

### Subjects and housing

Fourteen subadult–adult (20 months to 5 years of age at the time of the study) Goffin’s cockatoos (*Cacatua goffiniana;* formerly *goffini*), seven males and seven females, participated in this study. All of the birds were hand-reared by accredited German breeders and purchased with documentary evidence of origin and CITES papers. For individual identification, they were marked by colored leg bands. Previously, the whole group had participated in a number of cognitive experiments (see above).

The animals are housed together as a social group in an aviary consisting of an indoor part (45-m^2^ ground space, 3–6 m high, wall to gable) and an outdoor part (150-m^2^ ground space, 3–4.5 m high). The indoor part is enriched with wooden, free-hanging perches, artificial ponds, and wooden chew toys; the outdoor part is equipped with wooden, free-hanging perches and trees. During winter—October to May—the aviary is kept at 20 °C. Fresh drinking water and basic food (Australian Parrot Loro Parque Mix mixed with dried fruits) are available ad libitum, supplemented by two to three types of fresh fruit and various protein sources in the morning. The described housing conditions comply with the Austrian Federal Act on the Protection of Animals, and importantly, since this study was strictly noninvasive and based purely on behavioral tests, it was not classified as an animal experiment under the Austrian Animal Experiments Act.

### Behavioral monitoring

To record affiliative and agonistic behaviors, we conducted two cycles of behavioral observations, once a day between 9 am and 2 pm. We observed the group from outside the outdoor aviary four days a week during the summer (June to September 2012) and from outside the indoor aviary once a week during the winter (November 2012 to February 2013). Observations consisted of a 10-min focal per individual, throughout which time we recorded allopreening incidents and all displacements that occurred (see Table 2). Between every two focals, the nearest neighbor of each individual was documented. “Nearest neighbor” was defined as two or more individuals being within a range of 40 cm of one another. Overall, we recorded 4,090 min of observations (292 ± 36 min average per bird) and 245 nearest-neighbor records.

**Table 2:** List of social behaviors used to calculate the hierarchy and affiliations (affiliative behaviors that occurred <2 times in total were not included into the analysis)

Behavior	Description (Recorded Information)
*Unidirectional affiliative behavior*	
* Allopreening*	One bird touches the feathers of another bird with its beak for longer than 2 s (who touches whom). It often incorporates up and down or sideward movements of the beak through the plumage.
*Unidirectional agonistic behavior*	
* Mildly forced retreat*	One bird approaches another without physical contact, forcing it to retreat while producing defensive vocalization (who is the attacker, whom the recipient).
* Forced retreat*	One bird approaches another by engaging in physical contact, forcing it to retreat while producing defensive vocalization (who is the attacker, whom the recipient).
* Approach–retreat*	One bird approaches another, forcing it to silently retreat within 2 s (who is the attacker, whom the recipient).
* Threatened approach–retreat*	One bird approaches another, forcing it to silently retreat after being visually threatened^a^ within 2 s (who is the attacker, whom the recipient).

^a^A visual threat is generally indicated by extension of the crest and bill gaping (sometimes additionally with fluffing of the plumage and fanning of the tail). Physical contact is not achieved.

Observations during the summer were videotaped (JVC HD memory Camcorder, GZ-E10) through wire mesh, as well as voice-recorded (Sony Digital Dictation Machine, ICD-PX312). The observations during winter were conducted through a Plexiglas window (55 × 35 cm) inside the sliding door separating the experimental compartment from the indoor aviary, which prohibited qualitative video recording.

### Enhancement test

On the basis of the results from the behavioral observations, we selected a total of 12 pairs in three conditions: six dominance pairs, which were counterbalanced for sex (the rank difference between the paired subjects equaled at least three); three kinship pairs (individuals that had been hand-raised in the same nest box); and three affiliation pairs (based on the analysis below). Each bird served as both demonstrator and observer (subject).

To test for enhancement, we used a non-food-related object choice design. This design was chosen on the basis of the birds’ high motivation to interact with objects of all kinds. Behavioral observations showed that they spend a great amount of their daily activities interacting with different objects, and even fight over them (Auersperg et al., 2015; Szabo, 2013). Furthermore, previous experiments had highlighted their intrinsically structured object play (Auersperg, Oswald, et al., 2014; Auersperg et al., 2015).

**Fig. 1:**
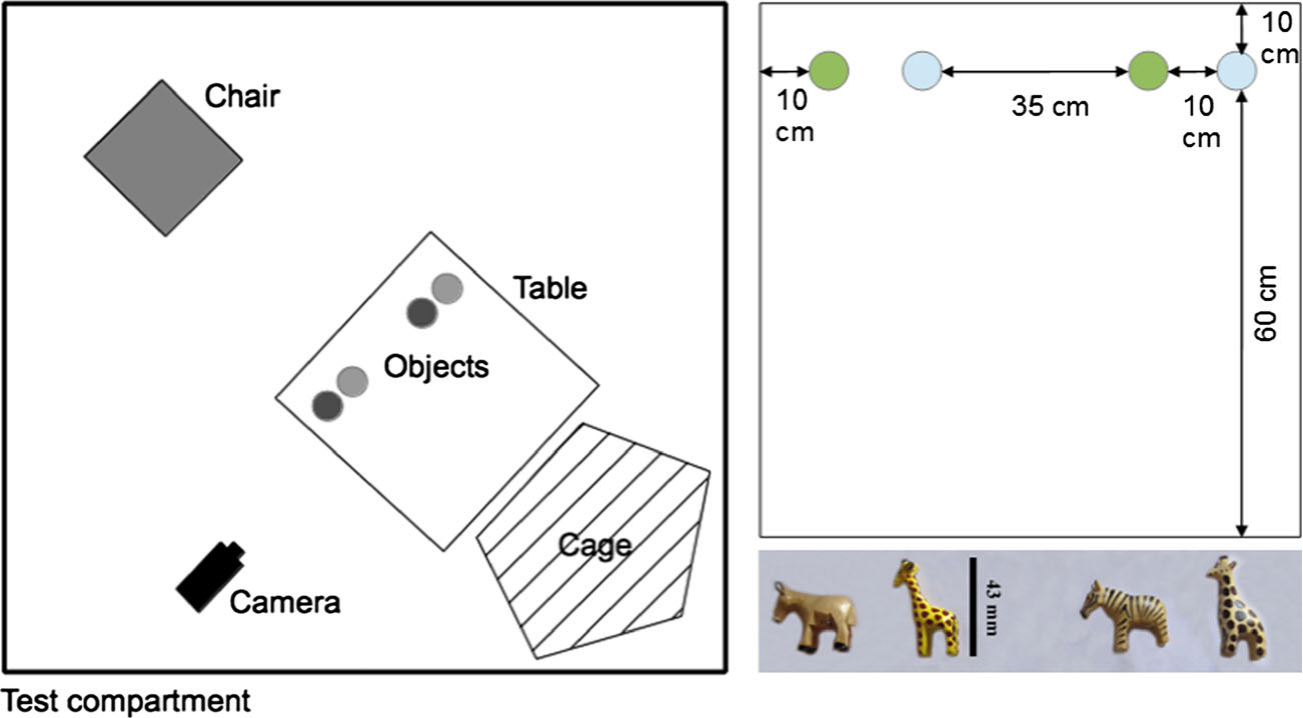
Simplified design of the enhancement test. (Left) Whole setup. (Right top) Object positions on the table. (Right bottom) Example of one of the object sets used during the test

Four objects (= set; 27 sets of objects, 108 objects total; size: minimum 25 mm, maximum 50 mm; material: wood, soft and hard plastic, metal; see the electronic supplementary material for further details) in two pairs were placed on a table. All items within a set were of the same approximate size and identical materials, but they varied in color and exact shape (see the electronic supplementary material): Each pair had some categorical similarities (e.g., both metal knights or wooden giraffes), but they did not look exactly the same (e.g., different knights, or one giraffe yellow and the other plain wood; see Fig. 1). Most of the objects (*N* = 94) were unknown to the subjects, although 14 of the objects were familiar to the group but were taken out of their usual context (i.e., small parts of wooden chewing toys they had encountered as a whole before, as well as two Kinder Surprise egg figures that had previously been used during animal training).

During the enhancement experiment (September 2012 to February 2013), every pair received up to ten trials (a maximum of one trial per day), switching demonstrator roles pseudorandomly so that each member of a pair demonstrated half of the total number of trials. Some birds showed higher levels of neophobia and were not forced to participate; consequently, one pair received only six trials, one pair five trials with only one bird demonstrating, one pair two trials, and one pair one trial (see Table 3). Each individual was tested only once per day, with at least one day between trials. To ensure constant motivation, we used each object set only once per animal. Trials were randomly selected from the pool of all possible trials scheduled for each pair.

**Table 3:** Name, sex, age, and rank position of each subject within the study group, as well as the dyads chosen within every enhancement test condition and the number of trials each pair participated in

Subject	Sex	Age (yrs)	Rank Pos.	Cond.	Partner	Trials	Pers. S.1	Pers. S.2	Pref. Test
Pipin	m	4	1	dom dom	Dolittle Olympia	5 5	.1	.4	.72
Figaro	m	5	2	dom dom	Konrad Fini	5 3^**^	.9	1.0	.31
Zozo	m	2,75	3	aff	Olympia	5	.9	.9	.47
Kiwi	m	2,75	4	kin	Heidi	5	1.0	.8	.81
Heidi	f	2,75	5	dom kin	Mayday Kiwi	5 5	.7	.9	.34
Konrad	m	2,75	6	dom aff	Figaro Lady	5 1	.6	.1	.42
Dolittle	m	1,75	7	dom kin	Pipin Mayday	5 5	.3	.4	.31
Muppet	m	2,75	8	kin	MoneyP.	5	.5	.5	.48
Money Penny	f	2,75	9	dom kin	Lady Muppet	1 5	0	.1	.35
Olympia	f	2,5	10	dom aff	Pipin Zozo	5 5	.5	.1	.18
Fini	f	5	11	dom aff	Figaro Pims	3^**^ 5	.2	.6	.45
Mayday	f	1,75	12	dom kin	Heidi Dolittle	5 5	1.0	.3	.43
LadyBird^*^	f	2,75	13	dom aff	MoneyP. Konrad	0 1	0	0	.38
Pims	f	4	14	aff	Fini	0	.9	1.0	.36

The last three columns show the probability of choosing the right side during perseverance test session one and two as well as during the preference test. ^*^Excluded due to motivation loss. ^**^Excluded due to pair formation.

#### Procedure

All tests were conducted on a plain, white table (1 × 1 m), with a parrot cage at one table end and the experimenter’s chair at the other (see Fig. 1). A trial started by visually isolating each pair from the group and carrying them into the test compartment. Thereafter, the experimenter placed the subject (the observer) into the large parrot cage, or some more neophobic birds on top of it, and the demonstrator on her shoulder. The demonstration phase began by placing a set of four objects on the experimenter’s end of the table, two on the left side and two on the right (see Fig. 1). Object placement was performed in full sight of both individuals. To control for enhancement effects caused by the experimenter, placement always started from left to right (from the observing subjects’ perspective). Due to the animals’ neophobia regarding large objects, we omitted the use of an occluder early during the experimental design.

**Fig. 2:**
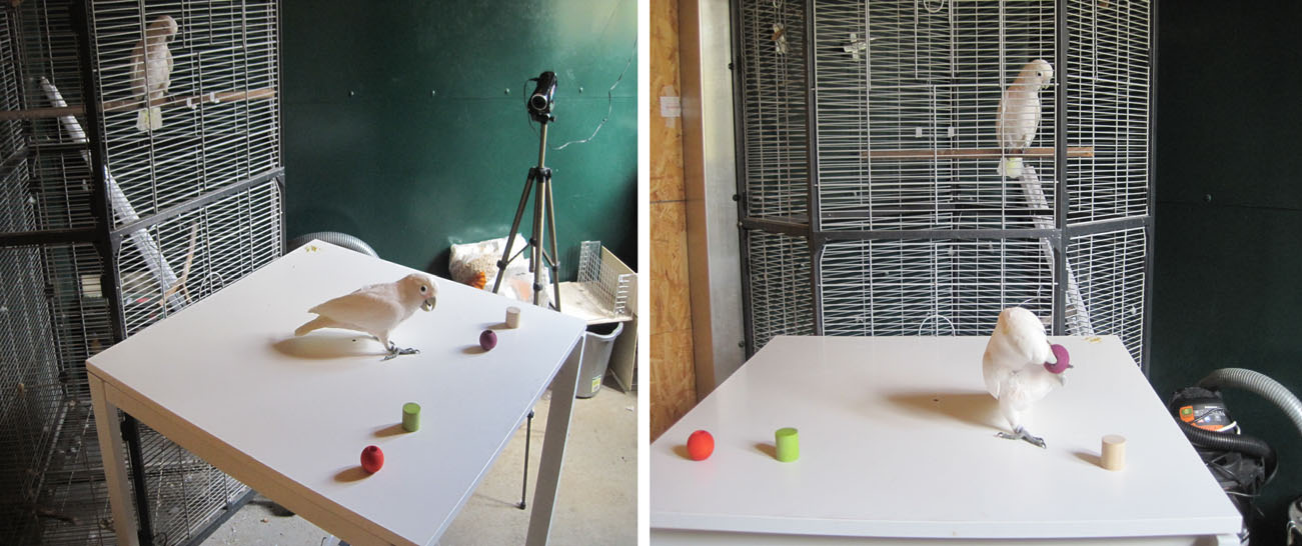
Testing situation during the demonstration phase in the experimental compartment. The subject is in the cage, observing the demonstrator on the table (left) and manipulating an object (right). The position of the video camera is visible in the left picture

Next, the experimenter released the demonstrator in the middle of the far end of the table, and it was allowed to pick up one item and manipulate it in full sight of the observer (see Fig. 2) for as long as it was interested in it, but not longer than 10 min. Furthermore, flying away with the object or losing it terminated the demonstration phase. If the demonstrator touched more than one object, the whole trial was terminated, and repeated a few days later using the same object set. As soon as the demonstrator let go of the object, the demonstration phase ended, and the animal was taken back to the group area.

The test phase began with the experimenter placing the items back on the table in the same order, again from left to right. The subject was then released from the cage and allowed to choose an item for itself. Trials were stopped if a bird did not touch any object for 15 min (unsuccessful trial). An animal would be excluded from the experiment after showing no motivation for an extended period of time, defined as five unsuccessful trials in a row. Therefore, one female (LadyBird) was excluded from testing because she lost motivation some weeks into the experiment. Another female (Pims) participated in only five trials because she could not be used as a demonstrator (she would not step on the table without seeing another bird step on it first). Furthermore, due to a pair formation during the mating season in February 2013, the data from one dominance pair (Figaro and Fini) were subsequently excluded from the analysis.

During testing, the experimenter wore mirrored sunglasses, avoided lateral head movements, and did not touch the birds or speak to them until the trial was over; furthermore, all trials were videotaped (JVC HD memory Camcorder, GZ-E10).

#### Data rating

If the subject chose an object of the same type but on the other side from the one chosen by the demonstrator, the choice was scored as “Different Side, Same Type” (DSST). Choosing an object on the same side but of a different type was scored as “Same Side, Different Type” (SSDT). Going for the same object as the demonstrator was scored as “Same Side, Same Type” (SSST), and choosing an object of a different type and on a different side was scored as “Different Side, Different Type” (DSDT).

For example, if the demonstrator manipulated the donkey (see the example object set in Fig. 1) during the demonstration phase, and the subject chose the zebra during the test phase, it was scored as DSST. However, choosing the yellow giraffe during the test phase would be scored as SSDT, choosing the donkey as SSST, and choosing the brown giraffe as DSDT.

### Perseverance test

We conducted two sessions of a perseverance test, before and after the enhancement experiment, to investigate any side preferences. Each session consisted of ten trials, with four to five months between test sessions. For this test, the experimenter placed two quarters of a cashew nut simultaneously on opposite sides of the table and then covered them, again simultaneously, with two identical pieces of paper (7 × 4.5 cm). Afterward, the subject was allowed to choose one side and consume the reward.

### Preference test

To look for any individual preferences among the items used, we conducted a preference test after the enhancement test. This order was chosen to ensure that subjects had high motivation to interact with the unfamiliar objects during the enhancement trials. Furthermore, since the pool of available objects was continuously expanded during the enhancement test, preference test sessions were conducted at the end of the experiment.

Each subject received a session of 12 trials for each set of objects used during its enhancement test trials. This was done from December 2012 to February 2013, at least once a day, three times a week. Due to the variation in trial numbers during the enhancement test, each bird received between three and 20 sessions, depending on the number of object sets presented during the enhancement test.

The four items were placed in a manner similar to the enhancement test procedure (from left to right, and in the same order), but equally spaced (about 15 cm between objects). Subsequently, the subject was allowed to choose one item and explore it for 1 min; the first object touched (with the bill or foot) was recorded.

### Statistical analysis

We used R Statistics, version 3.2.1 (R Core Team, 2016), for the statistical analysis. Additional packages used were modeest (Version 2.1; Poncet, 2012) to calculate the mode and lmerTest (Version 2.0-30; Kuznetsova, Brockhoff, & Christensen, 2016) to fit linear mixed-effect models (LMMs). Because our data violated the assumptions of parametric analysis, nonparametric tests were used, and all *p* values are two-tailed.

To determine the structure of the dominance hierarchy, we analyzed unidirectional agonistic behaviors (see Table 2) arranged in a matrix (actors in rows and recipients in columns) with MatMan 1.1, which hierarchically structured individuals from the highest- to the lowest-ranking bird (see Table 3). We observed no differences in recorded unidirectional agonistic behaviors (i.e., *displacements*; row-wise matrix correlation, *τ* = .495, *p* < .001), affiliative behaviors (*allopreening*; row-wise matrix correlation, *τ* = .342, *p* < .01), and nearest-neighbor records (row-wise matrix correlation, *τ* = .247, *p* < .01) between the first and second cycles of observations. Therefore, the respective records of both observational runs were used to calculate rank hierarchy and affiliated pairs. Furthermore, to analyze potential effects of sex on rank position, we applied Mann–Whitney *U* tests.

Affiliations (friendly relationships between two individuals) were determined by using the unidirectional allopreening and nearest-neighbor data. To find out whether close association is linked to socio-positive behavior in the same pairs, we looked for correlations between the allopreening and nearest-neighbor association data.

To identify enhancement effects, we fitted an LMM. As the target variable, we used the position chosen by the subject and included demonstrator choice position and condition (dominance, affiliation, or kinship) as the fixed effects. Demonstrator and subject identity were chosen as random effects. The residuals were normally distributed.

Individual side biases were analyzed using binomial tests measuring the probability of choosing the right side during perseverance and preference tests (see Table 3). To determine whether subjects’ side choices persisted over the course of the whole study, we applied a Wilcoxon signed-rank test and compared the numbers of right-side choices for each subject during the first and second sessions of the perseverance test. Additionally, we tested whether the demonstrators’ choice of pairs (pair: first and third or second and fourth objects) or sides (left, first and second; right, third and fourth object) had an influence on the subjects’ choices by fitting binomial generalized linear models (GLMs) with the pair–side choices of subjects as the target variable, and demonstrator choice, identity of demonstrator, and subjects as well as condition as fixed effects. After stepwise backward exclusion of terms, based on the Akaike information criterion (AIC), the simplest model was chosen.

We investigated object preferences using the mode. Incorporating the mode resulted in one value (in the range 1–4, indicating the position chosen most often) for each preference test session. In those instances in which an individual showed similar preferences for more than one object, these data were excluded from the analysis (NA; demonstrators: seven, subjects: eight sessions). We compared the modes with the objects chosen during enhancement. In both tests, the demonstrators chose the same object in 28 of 94 instances (29.8 %), and subjects in 27 of 94 (28.7 %).

To rule out experimenter-induced enhancement effects, we counted the number of times a demonstrator or subject chose a position in each condition during the enhancement test. The difference in the number of times the demonstrator or subject chose one of the four positions was analyzed using a Friedman test and a Wilcoxon signed-rank test post hoc.

Furthermore, to analyze whether the object handling times of the demonstrator had an effect on the subjects’ choices or whether they might be correlated with the presented object sets, handling time was determined using the video recordings, and we applied a GLM with the log-transformed handling times as the target variable and subjects’ choices, condition (dominance, affiliation, kinship), demonstrator and subject identity, and object set as fixed effects. Furthermore, we analyzed the possible interactions between demonstrator and subject identity and demonstrator identity and object set. After stepwise backward exclusion of terms based on the AIC, the simplest model was chosen.

On the basis of our methodology, we conducted a power analysis (G*Power version 3.1.9.2; Faul, Erdfelder, Lang, & Buchner, 2007) and calculated sample sizes, resulting in a distinguishable effect size between conditions.

## Results

### Behavioral monitoring

The subject group showed a significantly linear dominance hierarchy (row-wise matrix correlation, *h'* = .886, *p* < .001; de Vries, 1995, 1998), with males generally occupying higher-ranking positions than females (Mann–Whitney-test, *N* = 14, *Z* = −2.747, *p* < .01; see Table 3). This trend remained stable throughout the testing period (displacements: row-wise matrix correlation, *τ* = .495, *p* < .001). Within the hierarchy, we found no tied relationships (i.e., two birds showing equal numbers of agonistic behaviors; de Vries, 1995, 1998), but some inconsistencies (i.e., a relationship deviating from linearity; de Vries, 1995, 1998) were caused by one juvenile male (Dolittle).

Our analysis revealed a statistically significant correlation between allopreening incidents and the nearest-neighbor data (row-wise matrix correlation, *τ* = .231, *p* < .01), indicating that the birds engaged in these behaviors were affiliated. To determine affiliated pairs for the enhancement test, we used reciprocal allopreening as well as the nearest-neighbor frequencies (bidirectional nearest-neighbor frequencies above the third quartile); we identified a total of three pairs (see Table 3).

### Enhancement, perseverance, and preference tests

#### Enhancement

**Fig. 3:**
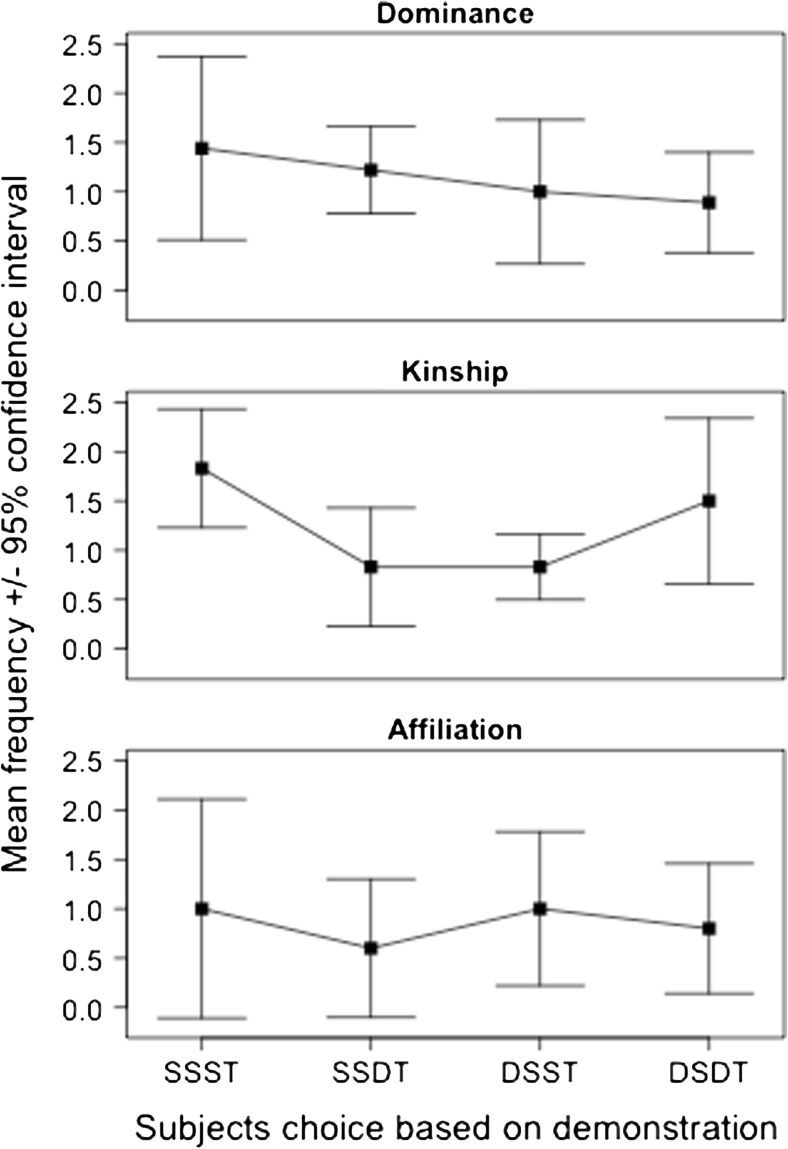
Mean frequencies and 95 % confidence intervals of subject choices based on demonstration, in the three tested conditions (dominance, kinship, and affiliation). SSST, same side, same type; SSDT, same side, different type; DSST, different side, same type; DSDT, different side, different type

All but one test subject (see the Method section) voluntarily participated during the test trials, and henceforth consistently chose objects for manipulation within the time given. Trials that had to be repeated because the demonstrator manipulated more than one object occurred mainly during the first two weeks of the experiment. Nine out of 56 trials (16 %) were repeated in the dominance condition, six out of 36 trials (17 %) in the kinship condition, and two out of 19 trials (11 %) in the affiliation condition. We failed to find an enhancement effect of demonstrator choice (LMM: estimate = −0.04142, *SE* = 0.12731, *df* = 10.07000, *t* = −0.325) or condition (LMM: estimate = −0.02328, *SE* = 0.19385, *df* = 75.03000, *t* = −0.120) on subject choices (see Fig. 3). The random effects demonstrator (LMM: *σ* ^2^ = .080199, *SD* = .28319, number of obs: 94, groups: subjects,14; demonstrator, 13) and subject identity (LMM: *σ* ^2^ = .003131 *SD* = .05596, number of obs: 94, groups: subjects,14; demonstrator, 13) explained only a very small part of the total variance.

#### Bias

Table 3 shows the probabilities of choosing the right side for each subject in both sessions of the perseverance test and throughout the preference test. A probability of 1.0 translates to choosing the right side on 100 % of trials, whereas as probability of 0.0 translates to choosing the left side on 100 % of trials. We found no difference between the numbers of right-side choices during Sessions 1 and 2 of the perseverance test (Wilcoxon signed-rank test: *N* = 14, *V* = 35, *p* = .8933).

During enhancement, only subject identity explained the subjects’ side choices (Subject Side Choice ~ Demonstrator Side Choice + Demonstrator + Subject + Condition: AIC = 105.64822, *df* = 21; Subject Side Choice ~ Subject: AIC = 94.67875, *df* = 13). The influence on pair choice was similar. On the basis of AIC, the model including only subject identity explained the data best (Subject Pair Choice ~ Demonstrator Pair Choice + Demonstrator + Subject + Condition: AIC = 122.3532, *df* = 21; Subject Pair Choice ~ Subject: AIC = 111.5110, *df* = 13).

#### Demonstrator behavior

We could find no influence on choices based on object placement during trials (Demonstrator: Friedman test, *N* = 4, *df* = 3, *χ* ^2^ = 7.9914, *p* = .04619; Subjects: Friedman test, *N* = 4, *df* = 3, *χ* ^2^ = 7.7105, *p* = .05239). Post-hoc analysis of the demonstrator choices revealed a significant preference for objects at Position 4 over Position 3 (Wilcoxon signed-rank test: *N* = 13, *V* = 7, *p* = .02096).

Demonstration time did not affect subject choices (GLM: estimate = 0.07756, *SE* = 0.11817; *t* = 0.656, *p* = .51493) independent of the condition (GLM: estimate = 0.37919, *SE* = 0.30561; *t* = 1.241, *p* = .22127). Stepwise backward exclusion resulted in elimination of the effects of subject position choice, subject identity, and condition, as well as the interaction between demonstrator identity and object set [log(Handling Time) ~ Subject Position Choice + Demonstrator + Subject + Object Set + Condition + Demonstrator × Subject + Demonstrator × Object Set: AIC = −5,013.984, *df* = 86; log(Handling Time) ~ Demonstrator + Object Set + Demonstrator × Object Set: AIC = −5,298.907, *df* = 86].

## Discussion

Our data suggest that the social group we observed is hierarchically organized and additionally is structured by affiliations: Individual cockatoos showed clear-cut relationships with each other. We failed to find any social enhancement effects of demonstrator choice during object manipulation. Instead, our tests showed a rapid formation of subject biases and individual preferences for specific object sets.

### Social relationships

Our analyses indicated that this groups’ dominance hierarchy was highly linear, with males occupying the top rank positions. Similar sex effects can be found in other social species, such as primates (e.g., Schino & Aureli, 2008) and corvids (e.g., Braun & Bugnyar, 2012; Chiarati, Canestrari, Vera, Marcos, & Baglione, 2010; Izawa & Watanabe, 2008). Although sexual dimorphism is not strongly expressed in the Goffin, the males are generally slightly bigger and heavier, with males weighing ~300 g and females ~250 g (Forshaw & Cooper, 2003). Furthermore, males tend to be bolder during novel object approach (at least within this study group; unpublished data). In most cockatoo species, the males are responsible for nest and territory defense (Forshaw & Cooper, 2003); therefore, it is likely that competition is greater between males than between females. Consequently, males show higher levels of aggression (Izawa & Watanabe, 2008), which may also explain why we could not identify any affiliated male–male pairs.

In theory, linear hierarchies are only stable in groups of ten or fewer individuals, and an increase in number is believed to result in inconsistencies (Drews, 1993; Jameson, Appleby, & Freeman, 1999; Kaufmann, 1993, cited by Chiarati et al., 2010). Accordingly, our focus group included 14 individuals, and, coherently, we found some minor inconsistencies related to one juvenile individual. Natural populations of Goffin’s cockatoos form large nomadic groups of hundreds of individuals during their juvenile–subadult period (Cahyadin et al., 1994); however, other reports from Singapore (Neo, 2012) have shown them foraging in smaller groups, indicating a kind of fission–fusion society. During this period, hierarchies could be useful within stable subgroups, whereas later, during adult life, the birds live in monogamous pairs or, seasonally, in family groups (Cahyadin et al., 1994).

### Social enhancement

So far, evidence on other avian species such as the kea (Gajdon, Fijn, & Huber, 2004; Heyse, 2012), common raven (Heyse, 2012; Schwab, Bugnyar, Schloegl, & Kotrschal, 2008), and carrion crow (Miller et al., 2014) suggests that local and/or stimulus enhancement plays a major role in these species’ object manipulations. In both ravens and kea, the amount of social learning increases when affiliated birds are present. Stimulus enhancement is most frequently shown in both species, and a correlation between local enhancement and rank position could be detected only in ravens (Heyse, 2012). Jackdaws, in contrast to ravens and kea, learned only in a foraging context, and more from nonaffiliates than from affiliated birds (Schwab, Bugnyar, & Kotrschal, 2008). Furthermore, free-ranging carrion crows were more prone to explore objects at the same location as conspecifics, indicating a preference for local, rather than stimulus, enhancement (Miller et al., 2014).

In this study, we tested enhancement in three social conditions (dominance, kinship, and affiliation), and contrary to previous findings in other large-brained birds, we found no significant effects on choices. It seems that (at least in this particular context) the Goffins’ explorative behavior remains largely uninfluenced by other group members. Although their manipulation of nonfood items is generally highly intense and intrinsically structured, as compared to many other large-brained birds (Auersperg et al., 2015), their ability to learn in an object choice task seems to take place more on an individual level. An alternative explanation might be found in the methodology. Generally, avian visual acuity is very high (Jones, Pierce, & Ward, 2007). Therefore, we can assume that our subjects were able to distinguish between the presented objects. However, their excellent vision might have led to rating the objects as four distinct items instead of pairs. Consequently, only choosing exactly the same object as the demonstrator (SSST) might have classified as enhancement. If this were the case, using identical objects would have enhanced our results. Nevertheless, because we usually find slight variability in shape and size between objects of the same category (e.g., pebbles, fruits, seeds) in natural situations, we deliberately chose to use similar rather than identical objects in this setup.

A recent study, conducted on the same sample of birds, revealed an effect of social learning in a tool-using task (Auersperg, von Bayern, et al., 2014). One innovative animal spontaneously started to manufacture tools and use them to fish for objects or food (Auersperg, Szabo, et al., 2012). This animal subsequently served as a demonstrator for other group members, and the results showed more progress in all members of the demonstration group than in a control group, which had only witnessed a set of magnetic controls. Ultimately, all males of the demonstration group succeeded in retrieving a food reward with a tool (Auersperg, von Bayern, et al., 2014).

It is possible that the Goffins’ motivation to learn socially about object affordances is substantially heightened when it becomes evident that food is involved. Because social learning was not food-rewarded in the present task, the direct benefit of the socially acquired information may not have differed from that of an asocial choice. Lefebvre and Palameta (1988) tested two groups of pigeons, in which one group observed a demonstrator pierce a paper cover to obtain a food reward from a well, whereas the other group observed the same motor task, but the demonstrators remained unrewarded. Only pigeons that had observed the rewarded demonstration learned the task. A behavior will only spread if it is better rewarded than its alternatives (Galef, 1992, 1995; Nicol, 1995). Although play behavior can be self-rewarding, this does not mean that a new behavior socially facilitated through play will always be favored over its alternatives. As in our study species, jackdaws (Schwab, Bugnyar, & Kotrschal, 2008) were uninfluenced by the choice of a demonstrator during an unrewarded object choice paradigm. However, jackdaws do select the same colored box previously chosen by a nonpreferred conspecific when food is involved.

Another alternative explanation for the contrasting results might be the effect of life experience on the ability to learn socially, due to an increase in age and brain development. Our observers were mostly subadults, and enhancement effects might appear at a later stage in their life. However, previous studies have revealed that ravens and kea are particularly prone to show enhancement with objects as juveniles and subadults (Heinrich, 1995; Heyse, 2012).

A sample of 14 cockatoos was available at the Goffin Lab to take part in this experiment. Nevertheless, for this particular study, a bigger sample size may have led to a clearer distinction between the tested social conditions. A power analysis (analysis of variance, *α* = .05) revealed that a total sample of 65 pairs would lead to an effect size of .5 and a power of .95. Using a nonparametric test (Wilcoxon signed-rank test, two tailed, *α* = .05), a sample size of 110 pairs in each condition would be necessary to get the same effect size and power. These results show the importance of choosing an appropriate sample size. However, in many studies involving the testing of birds and mammals, this is not always possible.

### Bias

Our analyses revealed strong individual tendencies to choose the same side and pair; furthermore, side choices remained constant over the course of several months. These findings could be linked to cerebral lateralization (Brown & Magat, 2011). Studies on other parrots have indicated left handedness (Brown & Magat, 2011; Friedmann & Davis, 1983). In contrast, our experiment showed that the preferences for left and right were more or less balanced within the group. Another explanation could be found, based on visual processing of information in the avian brain. A study by Brown and Magat (2011) suggested a link between the preferential use of one eye and handedness. Due to the lateral location of the eyes, preferred use of one eye could be responsible for attention being biased to that side, and consequently lead to a side bias in object choices (Brown & Magat, 2011). However, handedness and eye preferences have not yet been tested in this species.

Our results indicate the Goffin’s cockatoo’s playful and inquisitive nature (Auersperg, Oswald, et al., 2014; Auersperg et al., 2015). The effects of being confronted with new objects and the resulting eagerness to explore them may have overshadowed the influence of a demonstration by a conspecific, especially considering that the birds were encountering objects only briefly during the enhancement trials. Through our methodology, we aimed to take advantage of this eagerness to explore unfamiliar objects, and as a result we chose to test for preferences after testing for enhancement. However, had the subjects shown enhancement, post-hoc tests for preferences would not have had any informative value; in fact, they would have indicated a lack of enhancement. Nonetheless, our findings showed no enhancement, and therefore testing preferences afterward was not an issue in this case. Our finding concerning the influences of demonstrator identity and object set on the demonstrators’ handling times may be linked to behavioral syndromes in a way that specific colors are preferred over others (Eysenck, 1941).

The use of an occluder during the experimental setup was discarded, due to strong neophobia toward large screens in tight spaces. We could find no enhancement of ether the last or first object touched by the experimenter (experimenter-induced enhancement effects). However, demonstrators chose the fourth object more frequently than the third. This result could be linked to neophobia, considering the relative position of the fourth object within the test compartment (i.e., farthest away from any given wall).

In summary, our findings on social-group relationships are in accordance with previous finding in primates and corvids. Furthermore, non-food-type object exploration does not seem to be socially influenced. This suggests that individual preferences overpower the social influence of a demonstrating individual. Taking recent findings (regarding social learning of a tool-using task: Auersperg, von Bayern, et al., 2014) into account, a switch between social and asocial learning, depending on the benefits involved (Laland, 2004), seems to best explain these diverging results.

## Electronic supplementary material

Below is the link to the electronic supplementary material.

ESM 1 (PDF 5220 kb)
