# Supplementary material for: Within-group relationships and lack of social enhancement during object manipulation in captive Goffin’s cockatoos (Cacatua goffiniana)
Source: Learn Behav. 2016 Jul 12;45(1):7–19. doi: 10.3758/s13420-016-0235-0 (PMC5325860; doi:10.3758/s13420-016-0235-0)
Supplement: Supplementary file 1 — (PDF 5220 kb) [file 13420_2016_235_MOESM1_ESM.pdf]

Electronic Supplementary Material

Supplementary Table 1. Pictures, pair number, set identifier and description with size (longest side) in mm of all 108 objects used in the study.

Picture	Pair	Set	Description	Picture	Pair	Set	Description	Picture	Pair	Set	Description
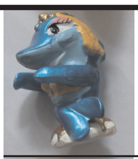	1	TS	Überra-schungsei® shark toy, hard plastic, 42mm	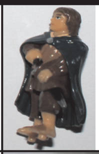	7	FF	Überra-schungsei® LotR toy, hard plastic, 49mm	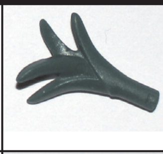	14	CP	Playmobil® plant wart, medium hard plastic, dark green, 23mm
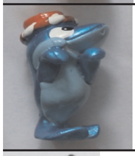	1	TS	Überra-schungsei® shark toy, hard plastic, 33mm	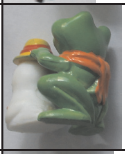	8	FF	Überra-schungsei® frog toy, hard plastic, 35mm	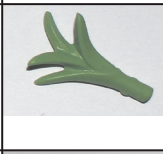	14	CP	Playmobil® plant wart, medium hard plastic, light green, 23mm
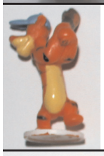 *1	2	TS	Überra-schungsei® Tigger toy, hard plastic, 36mm	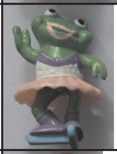	8	FF	Überra-schungsei® frog toy, hard plastic, 40mm	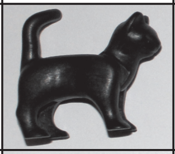	15	CM	Playmobile® cat toy, hard plastic, black, 36mm
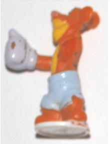 *1	2	TS	Überra-schungsei® Tigger toy, hard plastic, 42mm	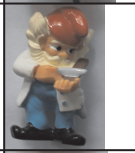	9	TD	Überra-schungsei® dwarf toy, hard plastic, 45mm	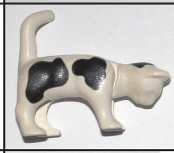	15	CM	Playmobile® cat toy, hard plastic, dots, 36mm
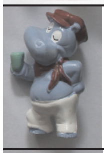	3	LH	Überra-schungsei® HappyHippo toy, hard plastic, 41mm	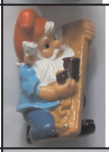	9	TD	Überra-schungsei® dwarf toy, hard plastic, 39mm	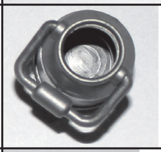	16	CM	Playmobile® milkcan toy, hard plastic, 25mm
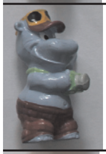	3	LH	Überra-schungsei® HappyHippo toy, hard plastic, 38mm	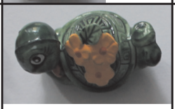	10	TD	Überra-schungsei® tortoise toy, hard plastic, 22mm	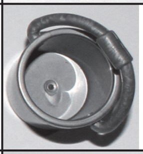	16	CM	Playmobile® bucket toy, hard plastic, 24mm
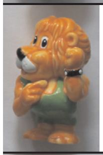	4	LH	Überra-schungsei® lion toy, hard plastic, 38mm	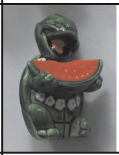	10	TD	Überra-schungsei® tortoise toy, hard plastic, 38mm	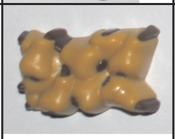	17	FP	Playmobile® food toy, hard plastic, yellow, 20mm
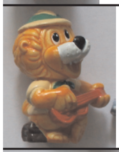	4	LH	Überra-schungsei® lion toy, hard plastic, 48mm	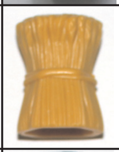	11	KH	Playmobile® hay toy, medium hard plastic, yellow, 35mm	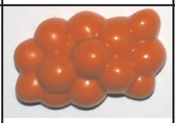	17	FP	Playmobile® food toy, hard plastic, orange, 20mm
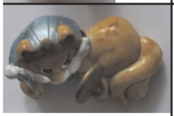	5	PC	Überra-schungsei® cat toy, hard plastic, 23mm	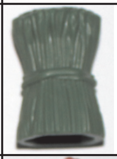	11	KH	Playmobile® hay toy, medium hard plastic, green, 35mm	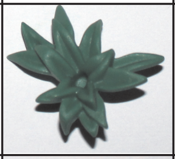	18	FP	Playmobile® plant toy, medium hard plastic, green, 32mm
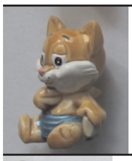	5	PC	Überra-schungsei® cat toy, hard plastic, 32mm	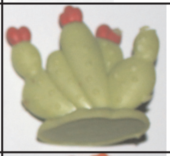	12	KH	Littlest Pet-Shop® cactus toy, soft plastic, green, 40mm	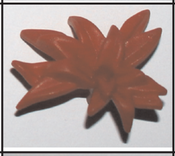	18	FP	Playmobile® plant toy, medium hard plastic, red, 32mm
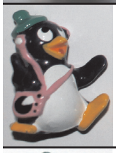	6	PC	Überra-schungsei® penguin toy, hard plastic, 50mm	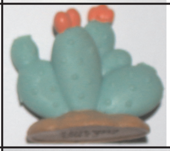	12	KH	Littlest Pet-Shop® cactus toy, soft plastic, torquoise, 40mm	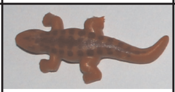	19	SG	Playmobile® salamander toy, hard plas-tic, red, 20mm
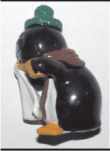	6	PC	Überra-schungsei® penguin toy, hard plastic, 45mm	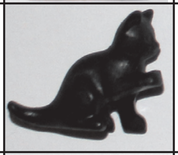	13	CP	Playmobile® cat toy, medium hard plastic, black, 29mm	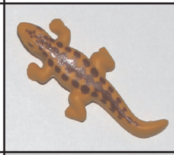	19	SG	Playmobile® salamander toy, hard plastic, orange, 20mm
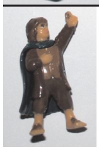	7	FF	Überra-schungsei® Fro-do LotR toy, hard plastic, 41mm	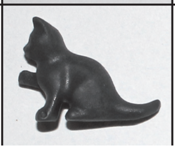	13	CP	Playmobile® cat toy, medium hard plastic, gray, 29mm	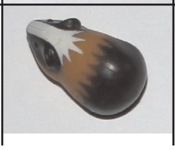	20	SG	Playmobile® guinea pig toy, hard plastic, 18mm

Picture	Pair	Set	Description	Picture	Pair	Set	Description	Picture	Pair	Set	Description
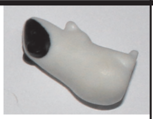	20	SG	Playmobile® guinea pig toy, hard plastic, 20mm	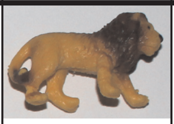	26	CL	Soft plastic lion toy, 25mm	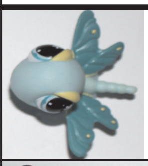	32	SD	Littlest Pet-Shop® dragonfly toy, soft plastic, green, 65mm
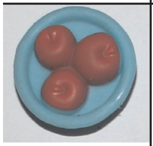	21	BF	Littlest Pet-Shop® food toy, apple, medium hard plastic, 22mm	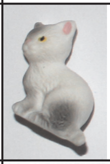	27	CC	Soft plastic cat toy, white, 20mm	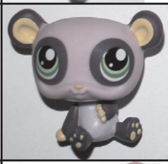	33	PB	Littlest Pet-Shop® panda toy, soft plastic, violet, 42mm
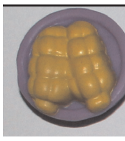	21	BF	Littlest Pet-Shop® food toy, corn, medium hard plastic, 21mm	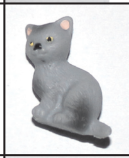	27	CC	Soft plastic cat toy, gray, 20mm	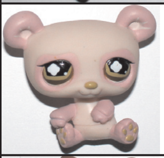	33	PB	Littlest PetShop® bear toy, soft plas-tic, pink, 42mm
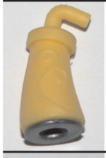	22	BF	Littlest Pet-Shop® bottle toy, medium hard plastic, yellow, 26mm	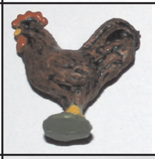	28	CC	Soft plastic chi-cken toy, brown, 32mm	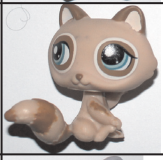	34	PB	Littlest Pet-Shop® racoon toy, soft plastic, brown, 50mm
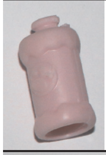	22	BF	Littlest Pet-Shop® bottle toy, medium hard plastic, pink, 29mm	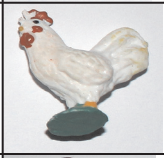	28	CC	Soft plastic chicken toy, white, 32mm	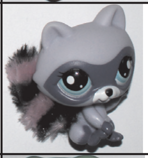	34	PB	Littlest Pet-Shop® racoon toy, soft plastic, blue, 50mm
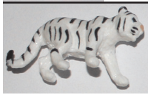	23	HC	Soft plastic tiger toy, 19mm	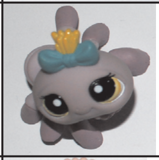	29	SS	Littlest Pet-Shop® spider toy, violet, soft plastic, 47mm	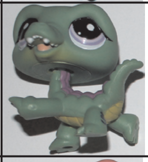	35	CA	Littlest Pet-Shop® crocodile toy, soft plastic, green, 50mm
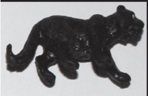	23	HC	Soft plastic pan-ther toy, 23mm	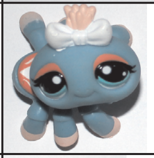	29	SS	Littlest Pet-Shop® spider toy, turquoise, soft plastic, 47mm	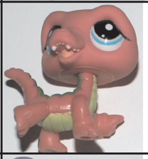	35	CA	Littlest Pet-Shop® crocodile toy, soft plastic, pink, 50mm
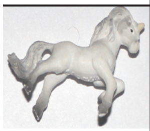	24	HC	Soft plastic horse toy, white, 32mm	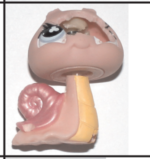	30	SS	Littlest Pet-Shop® snail toy, pink, soft plastic, 42mm	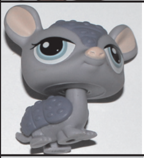	36	CA	Littlest Pet-Shop® armadillo toy, soft plastic, violet, 55mm
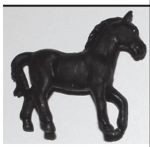	24	HC	Soft plastic horse toy, black, 34mm	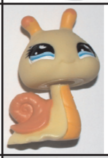	30	SS	Littlest Pet-Shop® snail toy, yellow, soft plastic, 50mm	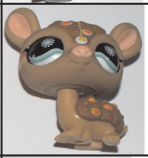	36	CA	Littlest Pet-Shop® armadillo toy, soft plastic, brown, 55mm
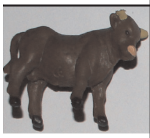	25	CL	Soft plastic cow toy, brown, 27mm	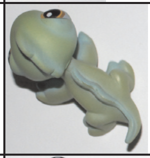	31	SD	Littlest Pet-Shop® saurian toy, soft plastic, green, 50mm	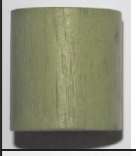 ∗2	37	ZB	Wooden cy-linder, green, 30mm
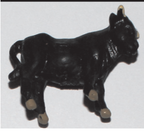	25	CL	Soft plastic cow toy, black, 27mm	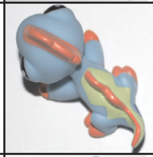	31	SD	Littlest Pet-Shop® saurian toy, soft plastic, blue, 50mm	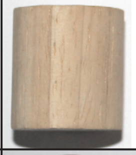 ∗2	37	ZB	Wooden cylin-der, plain, 30mm
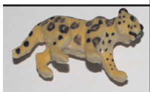	26	CL	Soft plastic leo-pard toy, 26mm	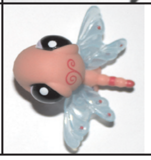	32	SD	Littlest Pet-Shop® dragonfly toy, soft plastic, pink, 65mm	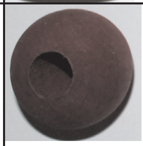 ∗2	38	ZB	Wooden ball, eggplant, 40mm diameter

Picture	Pair	Set	Description
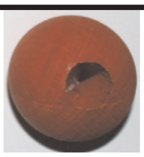 *2	38	ZB	Wooden ball, red, 40mm diameter
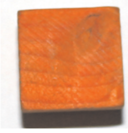 *2	*2 39	CT	Wooden cube, orange, 22mm
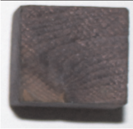 *2	39	CT	Wooden cube, violet, 22mm
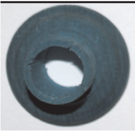 *2	40	CT	Wooden ring, green, 30mm
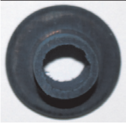 *2	40	CT	Wooden ring, blue, 30mm
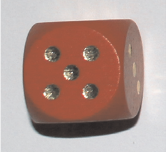	41	BD	Wooden dice, red, 20mm
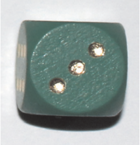	41	BD	Wooden dice, green, 20mm
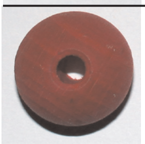 *2	42	BD	Wooden ball, red, 30mm diameter
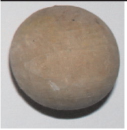 *2	42	BD	Wooden ball, plain, 30mm diameter
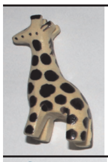	43	GD	Wooden giraffe, 42mm
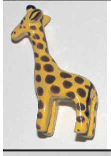	43	GD	Wooden giraffe, yellow, 36mm
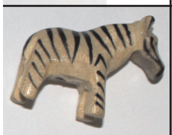	44	GD	Wooden zebra, 31mm
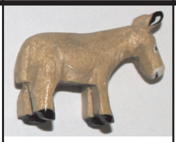	44	GD	Wooden donkey, 32mm
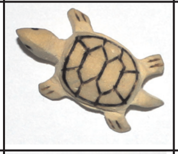	45	FT	Wooden turtle, painted, 30mm
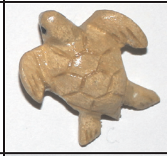	45	FT	Wooden turtle, plain, 35mm
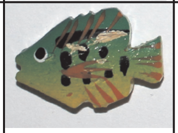	46	FT	Wooden fish, green, 22mm
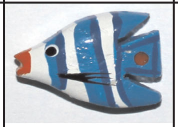	46	FT	Wooden fish, blua, 23mm
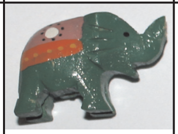	47	EB	Wooden elephant, green, 31mm
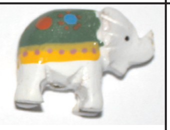	47	EB	Wooden elephant, white, 31mm
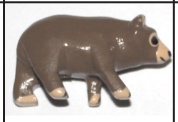	48	EB	Wooden bear, brown, 21mm
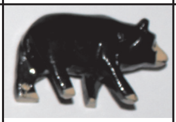	48	EB	Wooden bear, black, 21mm
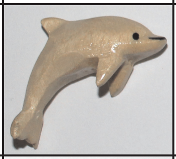	49	DW	Wooden dolphin, plain, 35mm
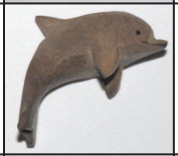	49	DW	Wooden dolphin, brown, 35mm
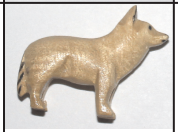	50	DW	Wooden wolf, 31mm
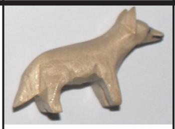	50	DW	Wooden fox, 31mm
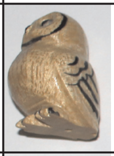	51	OF	Wooden owl, painted, 30mm
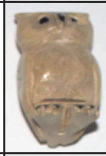	51	OF	Wooden owl, plain, 30mm
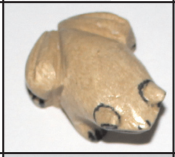	52	OF	Wooden frog, plain, 24mm
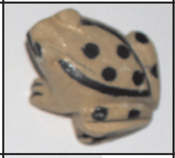	52	OF	Wooden frog, painted, 24mm
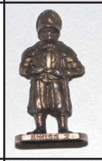	53	MM	Überraschunsei® knight toy, metal, 45mm
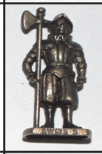	53	MM	Überraschunsei® knight with axe, metal, 45mm
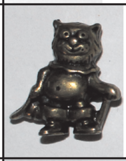	54	MM	Überraschungsei® Puss in Boots toy, metal, 30mm
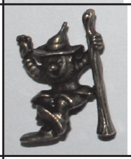	54	MM	Überraschungsei® Rupelstiltskin toy, metal, 30mm

*1 Toys previously used during animal training

*2 Part of wooden chewing-toys familiar to the subjects
